# From algorithms to action: twenty years of lessons from digital mental health and the future of human flourishing

**DOI:** 10.1177/17449871261468963

**Published:** 2026-08-20

**Authors:** Rhonda Wilson

**Affiliations:** Professor of Mental Health Nursing, School of Health and Biomedical Sciences, STEM College, RMIT University, Melbourne, VIC, Australia; Adjunct Professor, School of Nursing and Midwifery, University of Newcastle, Callaghan, NSW, Australia; Adjunct Professor, School of Nursing, James Cook University, Douglas, QLD, Australia; Adjunct Professor, Central Coast Local Health District, Gosford, NSW, Australia; Adjunct Professor, Central Coast Research Institute, Gosford, NSW, Australia

## Introduction

Over the past two decades, digital technologies have transformed mental healthcare, education and research. During this period, my work has spanned rural mental health nursing, telepsychiatry, implementation science, First Nations social and emotional well-being, workforce development and, more recently, artificial intelligence (AI). Looking back, the most important lessons have not been technological. Rather, they concern *access, relationships, culture, trust, implementation and human flourishing*. In this perspectives paper, I reflect on twelve lessons emerging from two decades of scholarship and leadership in digital mental health nursing. I argue that while AI offers significant opportunities, the future of digital mental health will depend on our ability to create systems that are *equitable, culturally safe, trustworthy and centred on human flourishing*.

When I first entered academic mental health nursing in the early 2000s, digital mental health was largely synonymous with computer-based cognitive behavioural therapy, early asynchronous internet interventions, informatic data bases and emerging electronic health records. AI remained largely the domain of science fiction (Higgins et al., 2023a). Few anticipated the scale of technological transformation that would follow.

Over the subsequent two decades, I worked across Australia, Denmark, Europe, New Zealand and international collaborations spanning rural mental health, telepsychiatry, implementation science, First Nations social and emotional well-being and digital mental health. Although technologies have changed dramatically, the central questions have remained remarkably consistent. *How do people access care? How do they build trust? How do systems adapt? How do individuals and communities flourish?*

**Figure 1.**
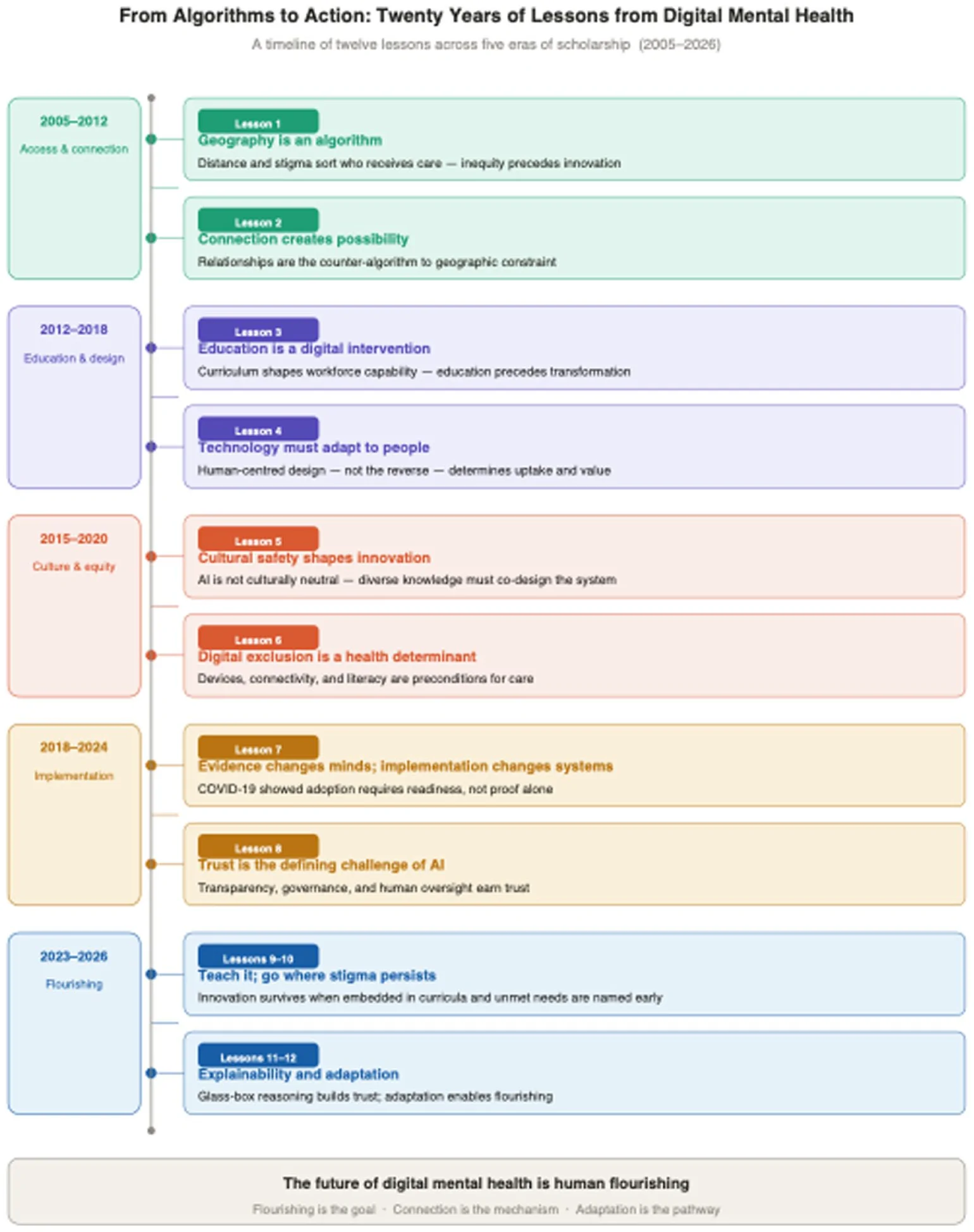
Twenty years of lessons from digital mental health scholarship (2005–2026).

Reflecting on this journey, outlined in Figure 1, I offer the perspective that the future of digital mental health is not fundamentally a technological challenge. It is a human one. The timeline maps 12 lessons across 5 interconnected eras: Access & connection (2005–2012); Education & design (2012–2018); Culture & equity (2015–2020); Implementation (2018–2024) and Flourishing (2023–2026). Lessons progress from recognising geography as a structural determinant of access (Lesson 1) through the relational, cultural and implementation foundations of digital health, to the contemporary challenges of trust, explainability and adaptation in the Al era (Lessons B-12). The overarching argument is that effective digital mental health is not primarily a technological challenge it is a human one.

## Twelve lessons from twenty years of digital mental health

### Lesson 1: Geography is an algorithm

My early scholarship and research focused on young people experiencing psychosis in rural and regional Australia (Wilson, 2007; Wilson and Usher, 2015; Wilson et al., 2012). Initially, I thought I was studying psychosis. In reality, I was studying *access.*

Long before AI emerged in mental healthcare, geography functioned as a powerful algorithm shaping who received care, when they received it and what outcomes they experienced. Distance, workforce shortages, stigma, social capital and service availability created unequal pathways to recognition, intervention and recovery. These experiences taught me that inequity often precedes innovation. Technology cannot solve access problems if access itself remains structurally constrained.

### Lesson 2: Connection creates possibility

Digital technologies first transformed my ability to connect with researchers, clinicians and communities beyond geographic boundaries (Wilson and Armstrong, 2016; Wilson et al., 2014). These experiences highlighted that digital transformation is not simply about interventions. It is also about relationships.

Connection creates possibility because knowledge, support and recovery are fundamentally relational processes. If geography functions as an algorithm that constrains access, connection becomes the counter-algorithm that creates opportunity. Communities of practice, professional networks and collaborative partnerships are themselves forms of digital infrastructure.

### Lesson 3: Education is a digital intervention

As online and blended learning expanded, I increasingly recognised that education itself functions as an intervention (Wilson and Hungerford, 2015). Every curriculum shapes future practice (Paliadelis et al., 2015). Every educational innovation influences workforce capability (Bembridge et al., 2025).

This insight informed my efforts to integrate digital mental health into nursing education, including early contributions to mental health nursing textbooks and postgraduate curricula (Hansen and Wilson, 2019; Procter et al., 2022). If healthcare systems require digital capability, then workforce development must precede digital transformation. Education remains one of the most powerful implementation strategies available to mental healthcare.

### Lesson 4: Technology must adapt to people

Work within telepsychiatry and European digital mental health programmes reinforced the importance of human-centred design. Early digital mental health research focused heavily on efficacy. However, usability, accessibility and user experience proved equally important (Sogaard Neilsen and Wilson, 2019).

Today, these ideas underpin contemporary discussions regarding co-design, personalisation and human-centred AI (Higgins et al., 2023a, 2024b; Wilson et al., 2023). Technology succeeds when it adapts to people. People should not be expected to adapt to technology.

### Lesson 5: Cultural safety shapes innovation

My work in First Nations social and emotional well-being profoundly changed how I understand innovation. Many of the challenges we seek to address are not technological; they are epistemological. They concern whose knowledge counts, whose voices are heard and whose experiences shape systems (Gregor et al., 2023).

As healthcare enters the AI era, these questions become increasingly important. AI systems are not culturally neutral. They reflect the assumptions, values and datasets upon which they are built. Cultural safety therefore requires moving beyond awareness to genuine partnership, self-determination and inclusion of diverse ways of knowing (Wilson and Waqanaviti, 2026; Wilson et al., 2024a, 2024b).

### Lesson 6: Digital exclusion is a social determinant of health

Digital participation is increasingly required to access healthcare, education, employment and social connection. Yet many individuals most likely to benefit from digital innovation remain excluded from it (Gregor et al., 2023).

Digital health does not exist separately from everyday life (Wilson et al., 2020, 2022, 2023, 2025). Before a person can engage with telehealth or digital therapeutics, they may require housing, income, education, a digital device, internet connectivity and digital confidence (Christensen et al., 2021; Chu et al., 2024). Consequently, digital exclusion is no longer merely a technology problem. It is a social determinant of health.

For mental health nursing, this raises important ethical questions regarding equity, accessibility and digital justice. The future challenge is not simply building better technologies but ensuring that everyone has the opportunity to benefit from them.

### Lesson 7: Evidence changes minds, implementation changes systems

One of the most enduring lessons from implementation science is that evidence alone rarely changes practice. Across telepsychiatry, internet-based interventions and large European implementation projects, successful adoption depended upon organisational readiness, leadership, workforce capability and local adaptation (Finch et al., 2024).

The COVID-19 pandemic provided a striking example. Innovations that had progressed slowly for years were implemented within months (Wilson et al., 2020). Telehealth became routine practice because systems adapted. As healthcare now navigates AI integration, the lesson remains unchanged. Evidence may change minds, but implementation changes systems.

### Lesson 8: Trust is the defining challenge of the AI era

The emergence of generative AI has accelerated discussions about the future of healthcare. Much attention has focused on capability. However, I would argue that the defining challenge of the AI era is trust.

Healthcare is fundamentally relational. Mental health nursing, in particular, relies on therapeutic relationships, empathy, transparency and accountability. Questions regarding bias, explainability, governance and safety are therefore not peripheral concerns. They are central implementation challenges (Higgins et al., 2023b, 2024a; Wilson and Higgins, 2023).

Trust cannot be engineered solely through technical sophistication. It must be earned through transparency, ethical governance and meaningful human oversight. Technologies succeed when people trust them, not simply when they work.

### Lesson 9: If you want innovation to survive, teach it

One of the most important lessons from my career is that innovation becomes sustainable not because it is effective, but because it is taught. Throughout my work, I have contributed to curriculum redesign, workforce development initiatives, accreditation activities and educational resources spanning digital mental health, telehealth and postgraduate mental health nursing education. Most recently, this work culminated in the development of a new textbook: *Digital Health Therapeutic Innovation: Clinical Practice for Nurses and Mental Health Professionals* (Wilson, 2026).

What became clear is that innovations rarely survive if they remain confined to research papers, pilot programmes or enthusiastic early adopters. They endure when they are embedded into curricula, professional standards, textbooks and continuing professional development.

The future mental health nursing workforce will require competencies that previous generations never anticipated (Wilson, 2018), including digital health, AI, disability nursing, integrated mental health and substance use care, gender-affirming practice and digital professionalism. Reviews in these areas consistently demonstrate that educational preparation often lags behind emerging practice demands (Bembridge et al., 2025; Higgins and Wilson, 2025; Hopwood et al., 2025; Hove et al., 2023; Jojo and Wilson, 2024).

Leadership therefore extends beyond research, policy and service delivery. It includes shaping what future practitioners learn. Curriculum decisions determine what knowledge is valued, what skills are prioritised and how prepared graduates are for contemporary practice.

Education remains one of the most powerful implementation strategies in healthcare. It creates workforce readiness before change becomes mandatory and ensures innovation is transferred from individual champions to entire generations of practitioners. For this reason, disciplinary leadership in mental health nursing education remains essential. Innovation survives when it becomes education.

### Lesson 10: Sometimes innovation begins where stigma exists

Another unexpected lesson is that innovation often emerges at the margins rather than the centre of healthcare conversations. Many topics that later become important areas of scholarship initially appear niche, controversial or insufficiently ‘important’ to attract attention.

Menopause was one such example. When our team began examining the experiences of nurses navigating menopause in the workplace, the issue was rarely discussed despite affecting a substantial proportion of the workforce. Our early studies identified significant gaps in organisational support and highlighted the psychosocial impacts of menopause on well-being, workforce participation and professional identity (Cronin et al., 2021, 2023).

Over time it became evident that menopause was not simply a women’s health issue. It was also a workforce, leadership, retention and equity issue. This work ultimately contributed to the development of the MIRROR Cycle, which emphasises reflection, self-awareness and organisational support as key elements of workforce well-being (Cronin et al., 2026).

The broader lesson extends beyond menopause. Many important healthcare innovations begin in areas characterised by stigma, silence or invisibility (Wilson et al. 2026a, 2026b). They emerge from populations whose experiences are overlooked and from problems others consider too difficult or peripheral to address.

Researchers and leaders therefore have a responsibility not only to follow trends but also to recognise unmet needs before they become widely visible. The issues ignored today may become the defining workforce and health challenges of tomorrow. Sometimes the most important innovations emerge where silence and stigma persist.

### Lesson 11: Explainability, transparency and trust

A defining challenge of contemporary healthcare is explainability. As AI becomes increasingly integrated into clinical practice, concerns regarding transparency, accountability and trust have become central (Higgins et al., 2024a, 2024b). The distinction between black-box and glass-box systems highlights a fundamental issue: stakeholders need to understand how conclusions are reached if they are to trust them.

Importantly, this challenge extends beyond AI. It also applies to qualitative research.

Both machine learning and qualitative inquiry involve moving from large volumes of complex data towards conclusions and recommendations. In both cases, confidence depends on whether the pathway from data to outcome can be understood and scrutinised.

This principle informs Empirically Textual Thematic Analysis (ETTA) (Gildberg and Wilson, 2023a, 2023b). ETTA was designed to make explicit the analytical pathway linking textual data, coding decisions, thematic development and final interpretation. By maintaining a transparent chain of evidence, the approach enables readers to understand how findings emerge from empirical data.

In this sense, ETTA represents a glass-box approach to qualitative inquiry. Rather than relying solely on researcher authority, it demonstrates the reasoning process underpinning analytical decisions.

Whether evaluating qualitative findings or algorithmic outputs, trust depends on transparency. Explainability therefore represents a shared methodological principle across both AI and qualitative research. Trust emerges when people can see how conclusions are reached.

### Lesson 12: Resilience is not enough; we need adaptation

For much of my career, resilience was one of the dominant concepts in mental healthcare. We spoke about resilient patients, resilient nurses and resilient organisations. While resilience remains important, I increasingly came to recognise that resilience alone is insufficient for addressing contemporary mental healthcare challenges (Francis et al., 2026; Margetts et al., 2024).

This insight emerged through my research in rural mental health, social capital and community well-being. The resulting *Rural Mental Health Ecology Framework* proposed that mental health is shaped by interactions between individuals, communities, services, environments and social systems (Wilson et al., 2015). Mental health was not simply an individual phenomenon; it was an ecological one.

This ecological perspective fundamentally altered how I viewed mental healthcare systems. Rather than linear structures, they appeared as complex adaptive systems – dynamic, interconnected and constantly evolving.

Subsequent work examining resilience, ecological theory and panarchy (Francis et al., 2026) reinforced this understanding. Healthy systems do not simply recover from disruption and return to a previous state. They adapt, reorganise and evolve.

The implications for mental healthcare are significant. Workforce shortages, digital transformation, climate change, AI and growing service demand are not isolated disruptions. They are ongoing pressures that continually reshape healthcare systems.

Resilience focuses on enduring disruption. Adaptation focuses on learning, responding, innovating and transforming in response to disruption. The distinction matters because many contemporary challenges are systemic rather than individual. Asking clinicians to become more resilient cannot substitute for organisational, professional and policy reform.

This perspective has become increasingly important in global mental health nursing workforce discussions. Sustainable reform requires resources, recognition, supportive environments and system-level change rather than reliance on individual coping alone (Wilson et al., 2026a, 2026b).

Ecology teaches that healthy ecosystems survive because they adapt. The same principle applies to professions, organisations and healthcare systems. Resilience helps us endure change, but adaptation allows individuals, communities and systems to flourish within it.

## Looking forward: From digital health to human flourishing

Reflecting across two decades of scholarship, I am struck by how often apparently unrelated projects were asking the same underlying question.

**Figure 2.**
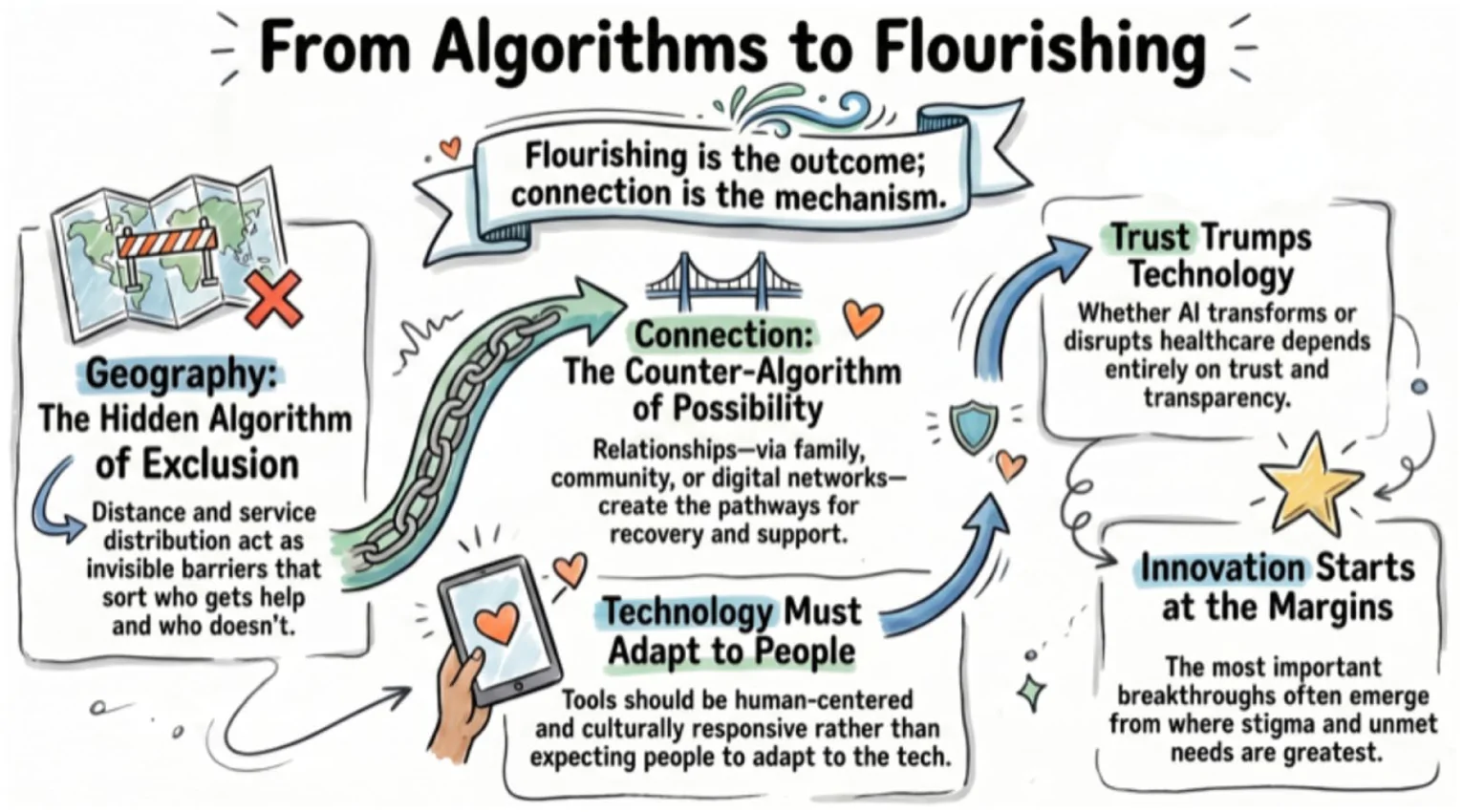
From algorithms to flourishing.

Psychosis, telepsychiatry, AI, menopause, First Nations well-being, implementation science and workforce development all appeared to be distinct topics. Yet each was ultimately concerned with *understanding how people flourish within complex systems* (Figure 2).

This perspective aligns with emerging ecological and complexity-informed approaches to mental healthcare, which increasingly recognise mental health systems as adaptive, interconnected and relational. Within such systems, human flourishing extends beyond symptom reduction. It encompasses belonging, purpose, connection, agency, cultural safety and the capacity to adapt.

The future of mental health nursing therefore requires moving beyond technological innovation alone. Priorities for the next decade include addressing digital inequities, strengthening trust in AI, embedding cultural safety within digital systems, supporting workforce capability and ensuring implementation approaches remain responsive to local contexts (Wilson et al., 2026a, 2026b). Most importantly, we must continue asking whether technological innovations genuinely enhance human well-being.

## Conclusion

Twenty years ago, I thought digital mental health was primarily about technology. Subsequently, I thought it was about access, implementation, workforce capability, culture and systems. Today, I believe these issues are inseparable.

AI, telehealth and digital innovations are powerful tools. However, they are not ends in themselves. Their value lies in whether they strengthen communities, improve well-being and support flourishing within increasingly complex mental health systems.

Ecology taught me that resilience helps us endure change, but adaptation is what allows individuals, communities, education and mental healthcare systems to flourish within it.

Mental health is not an individual problem occurring in isolation; it is an emergent property of complex, interconnected adaptive systems.

The future of digital mental health is not AI. *The future of digital mental health is ecologically informed human flourishing*.
